# Advances in Quantum Computing

**DOI:** 10.3390/e25121633

**Published:** 2023-12-08

**Authors:** Brian La Cour

**Affiliations:** Applied Research Laboratories, The University of Texas at Austin, Austin, TX 78758, USA; blacour@arlut.utexas.edu

Advances in quantum computing have continued to accelerate over the course of this Special Issue’s publication. In the past two years, we have observed major breakthroughs in hardware and algorithm development, as well as new, deep theoretical insights. In November 2022, IBM announced their record-breaking 433-qubit quantum computer, Osprey, with plans for developing a 100,000-qubit machine in the next ten years. In June 2023, the University of Science and Technology of China (USTC) first made available to global users their 176-qubit *Zuchongzhi* quantum computer, a successor to the *Zuchongzhi 2.1*, which they claim has a record quantum computational advantage of 1.0 × 10^8^ in sampling random circuits [1]. Shortly thereafter, in October 2023, USTC announced a breakthrough Gaussian boson sampling photonic experiment using their new *Jiuzhang 3.0* that boasts a quantum computational advantage of 1.5 × 10^16^ [2]. Meanwhile, in September 2023, PsiQuantum announced plans to build a one-million-qubit commercial photonic fusion-based quantum computer within the next six years.

This Special Issue has endeavored to capture some of the technical advances in this rapidly changing field. In Mingyoung Jeng et al.’s study, we find a novel method of producing depth-optimized circuits for performing multidimensional convolutions using a quantum computer (contribution 1). Brian García Sarmina and colleagues provide greater insight into the quantum approximate optimization algorithm by demonstrating that entanglement-based models possess an enhanced capacity to preserve information and maintain correlations over non-entangled models (contribution 2). Naya Nagy et al. propose a unique quantum honey pot scheme for detecting intruders using covert quantum sentinels to subtly detect when quantum information has been measured (contribution 3). Chen-Fu Chiang and Paul Alsing reconcile discrepancies between continuous-time quantum walk and adiabatic quantum computing optimization, with the latter option containing more structure than the former, using a modified catalyst Hamiltonian (contribution 4). Saad Darwish and co-workers describe a novel hybrid algorithm using semantic extraction and the quantum genetic algorithm to perform plagiarism detection, with simulations showing up to 20% improvement compared to classical genetic algorithms (contribution 5). Wenyang Qian et al. studied the use of the quantum approximate optimization algorithm (QAOA) to solve the traveling salesman problem, finding that well-balanced mixers usually outperform other QAOA mixer ansatzes (contribution 6).

From computing to communication, Kailu Zhang and colleagues propose an asymmetric measurement-device-independent quantum key distribution (MDI-QKD) protocol, the secure key rate of which is enhanced through advantage distillation (contribution 7). Sebastian Raubitzek and Kevin Mallinger investigate the applicability of quantum machine learning for classification and show that the variational quantum circuit and quantum kernel estimation methods perform better than basic machine learning algorithms (contribution 8). Jie Gao and co-workers consider enhanced quantum image encryption techniques, using a quantum DNA codec to enhance security and robustness (contribution 9). Manuel John et al. examine quantum kernel methods applied to quantum machine learning and develop several enhancements to provide significant performance improvements for several real-world classification tasks (contribution 10). Mark Goldsmith et al. study the use of quantum random walks to predict links within protein–protein interaction networks and demonstrate these walks’ ability to outperform classical random walks (contribution 11). Gabriele Cenedese and colleagues propose a method to efficiently generate random quantum circuits that result in high degrees of entanglement and use this as a benchmark for real-world quantum computers (contribution 12). Corey Trahan and colleagues demonstrate the application of hybrid quantum variational solvers to discrete solutions of partial differential equations, showing that they scale polylogarithmically based on the system size (contribution 13).

For business applications, Emanuele Dri et al. develop both new and generalized variants of current credit risk analysis quantum algorithms and test them using both simulated and real-world quantum computers (contribution 14). Similarly, Hanjing Xu and co-workers formulate an investment portfolio problem as a quadratic unconstrained binary optimization problem and solve a real-world example using D-Wave quantum annealers, resulting in optimized portfolios with more than an 80% return over classically derived ones (contribution 15). In contrast, Krzysztof Domino et al. use the D-Wave quantum annealer to solve real-world Polish railway network problems, finding that neither the 2000Q nor Advantage machines performed well in terms of solving these problems (contribution 16).

Congcong Feng and co-workers propose a new polar-based similarity metric for the K-nearest neighbor (KNN) algorithm and show that its use significantly improves performance, albeit only for the quantum version of KNN (contribution 17). Francisco Pereira and Stefano Mancini provide a general procedure for producing entanglement-assisted quantum-error-correction codes, providing better error protection compared to traditional stabilizer-based codes (contribution 18). Wenlin Zhao and co-workers propose a binary quantum neural network classification model based on an optimized Grover algorithm and partial diffusion, showing better performance over existing quantum neural network classification models (contribution 19). Thibault Fredon et al. perform spatial searches using a 2D grid with a coulomb potential and apply a discrete-time quantum random walk to demonstrate a quadratic scaling of the localization solution time that is robust against noise (contribution 20).

Sergey Tarasov and colleagues consider a Bose–Einstein condensate for studying quantum statistical phenomena and, in particular, how it might be used to perform Gaussian boson sampling (contribution 21). Andreas Burger and co-workers use a seven-qubit IBM quantum computer to perform digital quantum simulations of harmonically coupled spins and demonstrate the emergence of correlations (contribution 22). Abdirahman Alasow and colleagues study the use of Grover’s search algorithm to solve the maximum satisfiability problem and devise a novel quantum counter block within the standard oracle, demonstrating a significant reduction in the required number of ancilla qubits (contribution 23). Finally, Lihui Lv et al. use a variational quantum algorithm to solve the learning-with-errors problem and show a speed increase compared to classical solvers (contribution 24).

This Special Issue has shown the diversity of problems that can be solved using quantum computers, both existing noisy, intermediate-scale devices and future, fault-tolerant devices. With the recent rapid advances in quantum computing hardware, it should soon become apparent whether the promise of quantum computing anticipated in these works reflects the actual performance of future quantum computing technologies.

## References

[1] Zhu Q., Cao S., Chen F., Chen M.C., Chen X., Chung T.H., Deng H., Du Y., Fan D., Gong M. (2022). Quantum computational advantage via 60-qubit 24-cycle random circuit sampling. Sci. Bull..

[2] Deng Y.H., Gu Y.C., Liu H.L., Gong S.Q., Su H., Zhang Z.J., Tang H.Y., Jia M.H., Xu J.M., Chen M.C. (2023). Gaussian Boson Sampling with Pseudo-Photon-Number-Resolving Detectors and Quantum Computational Advantage. Phys. Rev. Lett..

